# Evaluation of Latent Tuberculosis Infection Risk in Liver Transplant Recipients

**DOI:** 10.3390/jcm15072803

**Published:** 2026-04-07

**Authors:** Miraç Öz Kahya, Serhat Erol, Dilara Kış Gökçecik, Elvan Onur Kırımker, Güle Çınar, Akın Fırat Kocaay, Deniz Balcı, Özlem Özdemir Kumbasar

**Affiliations:** 1 Department of Chest Diseases, Faculty of Medicine, Ankara University, 06620 Ankara, Türkiye; drserol@yahoo.com (S.E.); dilarakis44@gmail.com (D.K.G.); ozlemozdemir@yahoo.com (Ö.Ö.K.); 2 Department of General Surgery, Faculty of Medicine, Ankara University, 06620 Ankara, Türkiye; kirimker@ankara.edu.tr (E.O.K.); firatkocaay@gmail.com (A.F.K.); 3 Department of Infectious Diseases and Clinical Microbiology, Faculty of Medicine, Ankara University, 06620 Ankara, Türkiye; gbinjune@gmail.com; 4 Department of General Surgery, Faculty of Medicine, Bahçeşehir University, 34353 Istanbul, Türkiye; denizbalci1@yahoo.com

**Keywords:** tuberculosis, latent tuberculosis infection, liver transplantation, IGRA, TST

## Abstract

**Background/Objectives**: Tuberculosis remains one of the preventable causes of mortality among liver transplant recipients. The prevalence of tuberculosis in solid organ transplant recipients is higher than in the general population. The aim of this study was to evaluate the incidence of latent tuberculosis infection (LTBI) and active tuberculosis after liver transplantation. **Methods**: This is a retrospective, single-center, case–control study. Adult liver transplant candidates who were evaluated between 1 January 2016 and 31 December 2022 were retrospectively assessed. Patients with pre-transplant tuberculin skin test (TST) and/or interferon-gamma release assay (IGRA) results who underwent transplantation were included in this study. **Results**: A total of 111 liver transplant recipients with available IGRA and/or TST results were included; 70 were men (63.1%) and 41 were women (36.9%), with a mean age of 53.5 ± 11.3 years. Demographic, clinical, and laboratory characteristics were evaluated. The most common indication for liver transplantation was viral hepatitis (33.3%), followed by cryptogenic cirrhosis (19.8%) and hepatocellular carcinoma (10.8%). All patients had a Bacillus Calmette–Guérin (BCG) vaccination scar. Ten patients received grafts from deceased donors, while 101 underwent living-donor liver transplantation. No patient received LTBI treatment before transplantation, whereas LTBI treatment was initiated in four patients after transplantation. None of the patients had a diagnosis of active tuberculosis prior to transplantation. Thoracic computed tomography revealed findings compatible with tuberculosis sequelae in 11 patients (9.9%). During a median follow-up period of 49 [27–64] months after transplantation, no cases of active tuberculosis were observed among patients with positive TST and/or IGRA results. Patients were divided into two groups according to their TST and IGRA results. Group 1 consisted of patients with IGRA positivity and/or a TST ≥ 5 mm, while Group 2 included patients with a TST < 5 mm and negative IGRA results. The only statistically significant difference between the groups was the administration of LTBI treatment (*p* = 0.027); four patients in Group 1 received LTBI therapy. None of these patients were able to continue prophylaxis due to treatment-related adverse effects. **Conclusions**: Prophylaxis with hepatotoxic agents poses a substantial risk in liver transplant candidates. Since the hepatotoxicity may cause early cessation of LTBI treatment, the risk–benefit ratio of post-transplant LTBI therapy should be carefully assessed. In situations where LTBI treatment is deferred, close clinical monitoring is strongly recommended.

## 1. Introduction

Solid organ transplantation (SOT) is one of the routine therapeutic approaches that improves quality of life in patients with end-stage organ failure. The incidence of tuberculosis among solid organ transplant recipients has been reported to be 15–20 times higher, and tuberculosis-related mortality approximately 40% greater, compared with the general population [1,2].

Although donor-derived or re-infection tuberculosis has been reported, active tuberculosis mostly occurs due to reactivation of latent tuberculosis infection (LTBI). Thus, current guidelines recommend screening and treatment of LTBI in SOT patients.

Immunosuppression is a well-established risk factor for tuberculosis recurrence, with reported incidence rates ranging from 1.2% to 6.4% among transplant recipients worldwide and reaching up to 15% in endemic regions [1,3,4]. In Türkiye, the reported prevalence of tuberculosis among solid organ transplant recipients has been shown to range between 1.2% and 6.7% [5,6,7]. The tuberculin skin test (TST) and interferon gamma release assays (IGRAs) are the current available tests to evaluate an LTBI [8].

IGRAs based on T-cell responses represent a valuable alternative to the TST, demonstrating a high level of agreement between the two methods [9,10]. Some evidence indicates that QFT-G may have a stronger association with tuberculosis risk in immunocompromised individuals compared with TST; however, the available data are still limited, and both testing modalities have been associated with false-negative results [9,11].

According to current guidelines, individuals who are solid organ transplant recipients and have a positive TST and/or IGRA are recommended to undergo evaluation and be considered for LTBI therapy [4]. Türkiye is one of the endemic countries with a high risk of tuberculosis infection. Our tuberculosis screening protocol in liver transplantation candidates was a pre-transplant medical history for prior tuberculosis treatment or close contact with active tuberculosis, chest computed tomography (CT) and screening with TST or IGRA. In our national context, screening for LTBI in SOT candidates and recipients constitutes a fundamental component of pre-transplant evaluation and predominantly relies on the TST and/or IGRA [12]. Rapid IGRA-based platforms (e.g., iChroma-IGRA) and alternative skin test formulations (e.g., TST-Cyb TB) are not widely implemented in routine clinical practice. Candidates with LTBI should be given prophylaxis to prevent reactivation of disease in the setting of immunosuppression [13]. While LTBI treatment guidelines do not differentiate by organ type, tuberculosis epidemiology varies across SOT recipients. Although liver transplant recipients exhibit a lower prevalence of active tuberculosis compared to other solid organ transplant recipients, they demonstrate a higher susceptibility to hepatotoxicity associated with LTBI treatment [4]. Therefore, a thorough evaluation of LTBI treatment is particularly warranted in liver transplant recipients.

The present study aimed to assess the presence of LTBI in liver transplant recipients and to discuss LTBI treatment in these patients.

## 2. Materials and Methods

This is a retrospective observational study including patients aged 18 and higher who underwent liver transplantation at the Ankara University School of Medicine Transplantation Center from 1 January 2016 to 31 December 2022. We reviewed electronic files and national dispensary records of patients. Our local ethics committee approved this study (ethic approval number: İ09-843-25).

We collected patients’ data from electronic medical records: demographic features, smoking status, the etiology of transplantation, information regarding previous TB and LTBI diagnosis and treatment, and chest CT reports; radiological findings associated with LTBI (apical fibronodular lesions, calcified solitary nodules, calcified lymph nodes, or pleural thickening), and TST and IGRA results were also recorded. A TST result was considered positive when the diameter of the indurate area was ≥5 mm 48–72 h after intradermal injection of purified protein derivative. The threshold value of ≥5 mm for TST positivity was selected based on established international guidelines for high-risk populations, including immunosuppressed individuals such as transplant recipients and patients receiving biological therapies. This cutoff reflects the increased susceptibility to tuberculosis reactivation in these groups and is widely accepted in clinical practice. IGRA testing was performed in cases where TST was available. In this study, a total of two patients with indeterminate IGRA results and 19 patients who had no TST and IGRA results were excluded from the analysis.

### Statistical Analysis

Data distribution was assessed for normality using appropriate statistical tests (e.g., Shapiro–Wilk or Kolmogorov–Smirnov tests), as applicable. Continuous variables were expressed as mean ± standard deviation or median (interquartile range) according to their distribution, and comparisons were performed using appropriate parametric (independent samples *t*-test) or non-parametric (Mann–Whitney U test) methods. Categorical variables were summarized as frequencies and percentages and compared using the chi-square test or Fisher’s exact test, as appropriate. The statistical analysis of data was carried out using SPSS V.27 (SPSS Inc., Chicago, IL, USA). The significance level was considered to be *p* < 0.05.

## 3. Results

**Figure 1:**
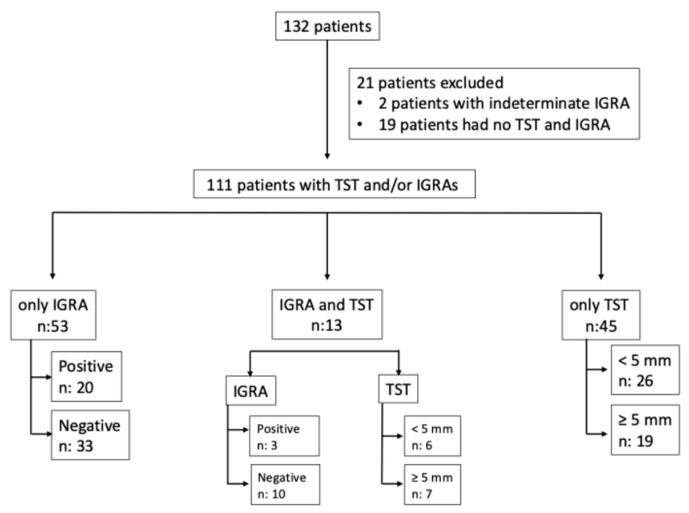
Flow chart of study.

A total of 132 patient received a liver transplant during the study period. Nineteen patients who had no TST and/or IGRA results were excluded. Two patients were also excluded because of indeterminate IGRA results (Figure 1). A total of 111 liver transplant recipients with available IGRA and/or TST results were evaluated. Among these patients, there were 70 men (63.1%) and 41 women (36.9%), with a mean age of 53.5 ± 11.3 years.

Demographic, clinical and laboratory findings of the patients are summarized in Table 1.

The most common indication of liver transplantation was viral hepatitis (33.3%), followed by cryptogenic cirrhosis (19.8%) and hepatocellular carcinoma (10.8%). Sixty-four percent of patients were transplanted because of other indications. All patients had scar of Bacillus Calmette-Guerin (BCG) vaccination on their arms. These included 10 cadaveric transplant recipient and 101 living-donor transplant recipients. LTBI treatment was not recommended to any of the liver transplantation candidates. There was no patient diagnosed with active tuberculosis infection before transplantation.

Findings compatible with TB sequelae were observed on thorax CT scans in 11 patients (9.9%). No patient received anti-tuberculosis prophylaxis prior to transplantation.

During a mean follow-up period of 49 [27–64] months following liver transplantation, no cases of active tuberculosis were detected among patients who had a positive TST and/or IGRA result prior to transplantation. Three patients were evaluated for active tuberculosis suspicion after transplantation, such as tuberculosis lymphadenitis and abdominal tuberculosis. Active tuberculosis was ruled out with microbiological and pathological examinations.

All patients included in this study underwent baseline screening for LTBI used either with TST, IGRA, or both, depending on clinical practice patterns and test availability during the study period. Due to the retrospective design, not all patients had complete dual-test data. Patients without available TST and/or IGRA results were excluded from the analysis to ensure a reliable assessment of LTBI status. Patients were categorized into two groups based on their TST and IGRA results (Table 2). Group 1 included patients with evidence of latent TB infection, defined as a positive IGRA and/or a TST induration of ≥5 mm, whereas group 2 consisted of patients who did not require LTBI treatment. Among the thirteen patients in whom both TST and IGRA were performed, seven were classified into group 1, defined as the group requiring LTBI treatment. Among the seven patients with a TST induration of ≥5 mm, three also had a positive IGRA result. The only statistically significant difference between the groups was the administration of LTBI treatment (*p*: 0.027). In Group 1, four patients received therapy for latent tuberculosis infection.

Among 111 liver transplant recipients, only four patients received latent tuberculosis treatment after transplantation. Clinical, laboratory and outcome results of these patients are shown in Table 3. These patients received Isoniazid (INH) prophylaxis after liver transplantation. Treatment of LTBI was with INH at a dose of 5–10 mg/kg/d, with a maximum dose of 300 mg per day. INH is currently initiated in the post-transplant period, after liver enzyme stabilization. However, none of the patients completed the treatment due to increase liver enzymes and gastrointestinal symptoms such as nausea and abdominal distension.

## 4. Discussion

In the current study, conducted in a tuberculosis-endemic region, none of the liver transplant patients with LTBI developed active tuberculosis even without treatment or early cessation due to side effects.

LTBI treatment is recommended in patients with positive IGRA and/or TST results, as well as for individuals who met epidemiological or clinical–radiological criteria even when TST or IGRA results were negative. These criteria were defined as the presence of stable, untreated tuberculosis lesions on imaging, a history of previously incomplete tuberculosis treatment, or recent close contact with an individual diagnosed with active tuberculosis [8,14,15,16].

The TST and IGRAs are currently available tools for the evaluation of LTBI; however, their positive predictive value for predicting the development of post-transplant tuberculosis in solid organ transplant recipients is limited [8]. In the Spanish RESITRA cohort, 4388 patients who underwent solid organ transplantation were evaluated, and the incidence of tuberculosis was found to be 27 times higher than that in the general population; age and lung transplantation were identified as the only independent risk factors for post-transplant tuberculosis [14,17]. In endemic regions such as Africa, the Middle East, and Asia, the incidence rates are even higher [14]. Effective prevention, prompt diagnosis, and appropriate treatment are essential components for the optimal monitoring of transplant recipients with respect to tuberculosis. Both the Spanish RESITRA cohort and a large Korean study including 57,103 patients demonstrated that, in non-endemic regions, the risk of developing tuberculosis after solid organ transplantation is highest within the first year following transplantation [17,18]. In our study, the median duration of post-transplant follow-up was 49 months. Patients were followed for a sufficient period to adequately detect the development of tuberculosis.

LTBI treatment was recommended for patients with positive IGRA and/or TST results, as well as for individuals who met epidemiological or clinical–radiological criteria. These criteria were defined as the presence of stable, untreated tuberculosis lesions on imaging, a history of previously incomplete tuberculosis treatment, or recent close contact with an individual diagnosed with active tuberculosis [15]. According to the American Thoracic Society, a TST induration of ≥5 mm is considered positive; however, higher cut-off values may be applied in countries with a high incidence of tuberculosis. In anergic patients, tuberculosis may develop despite a negative TST result. Therefore, prophylactic treatment is recommended in the presence of clinical or radiological findings suggestive of untreated tuberculosis or a history of close contact with patients with active tuberculosis [14,19]. In a study conducted by Kim et al. involving 312 kidney transplant recipients, positive IGRA results were shown to predict the risk of tuberculosis in kidney transplant candidates with negative TST results [20]. In another study involving 398 lung transplant recipients, the TST result was not identified as a risk factor for the development of tuberculosis [21]. Therefore, the TST has significant limitations, and a positive TST is a weak marker for post-transplant tuberculosis. Consequently, IGRAs have increasingly been used as an alternative to the TST in this setting. However, the low performance of IGRA in solid organ transplant recipients has also been reported, including in the liver transplant study by Hand et al. [22]. This may be related to the high level of immunosuppression in these patients. Negative TST results and indeterminate IGRA results complicate the assessment of the risk of progression to tuberculosis. Given the limited reliability of TST and IGRA when used alone, performing both tests in patients at risk may provide more reliable results. In our study, patients who underwent both tests were included.

Hepatotoxicity due to LTBI treatment is a major concern, especially for liver transplant recipients who already have vulnerable liver function. It may be difficult to distinguish drug-induced hepatotoxicity from other complications like acute rejection. In a study conducted in Korea, 28% of liver transplant programs reported that they do not perform screening or provide treatment for LTBI. The potential hepatotoxicity of anti-tuberculosis medications and the high prevalence of LTBI are reported as the probable reasons [23]. In SOT recipients, the management of LTBI requires a highly individualized approach that reflects contemporary best practices. The choice of preventive therapy should be guided by multiple interrelated factors, including baseline organ function—particularly hepatic reserve—anticipated intensity of immunosuppression, and the risk of clinically significant drug–drug interactions. Whenever feasible, initiation of LTBI treatment in the pre-transplant period is preferred, as it allows for safer administration and minimizes the complexity of post-transplant pharmacological management. In the posttransplant setting, treatment decisions must be carefully balanced against the risks of hepatotoxicity and interactions with immunosuppressive agents, especially calcineurin inhibitors. Regimen selection should therefore be tailored on a case-by-case basis, integrating clinical status, timing relative to transplantation, and the overall risk of tuberculosis reactivation. This individualized strategy is essential to optimize both patient safety and graft outcomes.

In our cohort, detailed clinical evaluation of patients receiving isoniazid provided important insight into hepatotoxicity risk. Liver enzyme elevations were observed as mild-to-moderate increases in alanine aminotransferase and aspartate aminotransferase, with peak values ranging from approximately two to five times the upper limit of normal (ULN) [12]. In line with established clinical practice, isoniazid was discontinued in patients who developed transaminase elevations exceeding 3× ULN in the presence of symptoms or 5× ULN in asymptomatic individuals, and no cases of severe liver failure were observed. Notably, treatment discontinuation due to hepatotoxicity, including intolerance in a subset of patients, may be partly explained by advanced age, which is a recognized risk factor for isoniazid-related liver injury and should be considered when planning LTBI therapy. These findings, together with the observed challenges in treatment tolerability, have led to a shift in our institutional approach: post-transplant LTBI treatment is no longer routinely administered in liver transplant recipients, and patients are instead closely monitored for the development of active tuberculosis. In clinical practice, LTBI treatment in solid organ transplant candidates and recipients remains largely based on isoniazid-containing regimens, which are widely adopted due to established efficacy, clinician familiarity, and relative ease of integration into transplant workflows. Although shorter rifamycin-based regimens, such as 3HP and 4R, have gained increasing support in international guidelines, their real-world uptake in transplant populations remains limited. This is primarily driven by concerns regarding clinically significant drug–drug interactions, particularly with calcineurin inhibitors and other immunosuppressive agents, which may compromise graft function and require complex dose adjustments. In addition, uncertainties related to tolerability and the feasibility of implementing these regimens within the peri-transplant period further restrict their use. Consequently, treatment decisions in this population remain highly individualized, balancing the risk of tuberculosis reactivation against potential treatment-related toxicity and pharmacological interactions.

The management of LTBI in SOT recipients necessitates a structured multidisciplinary care model to ensure both safety and effectiveness. Close collaboration among transplant hepatology/surgery, infectious diseases specialists, clinical pharmacists, nursing teams, and tuberculosis control or public health programs is essential throughout the continuum of care. This integrated approach is particularly critical for the early detection and monitoring of hepatotoxicity, proactive management of clinically significant drug–drug interactions with immunosuppressive agents, and optimization of treatment adherence and completion. In addition, coordinated patient education, standardized monitoring protocols, and clear communication between disciplines contribute to improved clinical outcomes. Such a multidisciplinary framework is fundamental to balancing the risks of tuberculosis reactivation against treatment-related toxicity, while simultaneously safeguarding graft function and overall patient prognosis.

This study has several limitations. First, its retrospective design and single-center setting may cause selection bias. Additionally, the relatively small sample size limits the statistical power of the analysis. Therefore, the generalizability of the findings to the broader population remains limited.

## 5. Conclusions

In liver transplant recipients, clinicians should remain vigilant regarding the potential hepatotoxicity associated with LTBI treatment regimens. The necessity of prophylactic therapy should be carefully evaluated, and prophylaxis indications should be reconsidered comprehensively in patients who are not at high risk for progression to active tuberculosis. In many cases where LTBI treatment is indicated, therapy is avoided due to additional clinical concerns. Prophylaxis with hepatotoxic agents poses a substantial risk in liver transplant candidates. Because LTBI treatment may need to be discontinued due to elevations in liver enzyme levels, the risk–benefit balance of post-transplant LTBI therapy should be carefully assessed. In situations where LTBI treatment is deferred, close clinical monitoring is strongly recommended. This is a retrospective, single-center study with a relatively small sample size. Thus, generalization of results to population would not be appropriate.

## Figures and Tables

**Table 1:** All patients’ demographic, clinical, and radiological findings.

	All Patients n: 111
Age, years ± SD	53.5 ± 11.3
Gender, male, n (%)	70 (63.1)
Transplantation type, n (%)	
Cadaveric transplantation	10 (9.1)
Living donor	101 (90.9)
Indication, n (%)	
Alcohol	11 (9.9)
Viral hepatitis	37 (33.3)
Autoimmune hepatitis	9 (8.1)
Hepatocellular carcinoma	12 (10.8)
Toxic hepatitis	1 (0.9)
Wilson’s disease	5 (4.5)
Non-alcoholic steato hepatitis	4 (3.6)
Hydatid cyst	2 (1.8)
Congenital hepatic fibrosis	1 (0.9)
Primary biliary cholangitis	3 (2.7)
Cryptogenic cirrhosis	22 (19.8)
Primary sclerosing cholangitis	4 (3.6)
Follow-up period, months, median [IQR]	49 [27–64]
TST, n (%)	
0–4 mm	32 (28.8)
≥5 mm	26 (23.4)
IGRA, n (%)	
Positivity	23 (20.7)
Negativity	43 (38.7)
Chest computed tomography, n (%)	
Fibrotic changes	11 (9.9)
Active lesions	0
Diagnosis of tuberculosis before LT, n (%)	0
Latent tuberculosis treatment after LT, n (%)	4 (3.6)
Nodules on CT after LT, n (%)	52 (46.8)
Exitus, n (%)	14 (12.6)

SD: standard deviation, n: number, TST: tuberculin skin test, IGRA: interferon-gamma release assay, LT: liver transplantation, CT: computed tomography.

**Table 2:** Comparison of groups according to TST and IGRA results.

	Group 1 n: 46	Group 2 n: 65	*p* Value
Gender, male, n (%)	30 (65.2)	40 (61.5)	0.842
Age, years ± SD	56.5 ± 10.2	52.1 ± 12	0.118
Transplantation type, n (%)			
Cadaveric transplantation	7 (15.2)	3 (4.6)	0.089
Living donor	39 (84.8)	62 (95.4)	
Indication, n (%)			0.284
Alcohol	7 (15.2)	4 (6.2)	
Hepatitis	18 (39.1)	19 (29.2)	
Autoimmune hepatitis	3 (6.5)	6 (9.2)	
Hepatocellular carcinoma	4 (12.1)	6 (9.2)	
Wilson’s disease	2 (4.3)	3 (4.6)	
Non-alcoholic steato hepatitis	0	4 (6.2)	
Hydatid cyst	0	2 (3.1)	
Congenital hepatic fibrosis	0	1 (1.5)	
Primary biliary cholangitis	1 (2.2)	2 (3.1)	
Cryptogenic cirrhosis	8 (17.4)	14 (21.5)	
Primary sclerosing cholangitis	0	4 (6.2)	
Toxic hepatitis	1 (2.2)	0	
Follow-up period, months, median [IQR]	48 [31.5–62.2]	49 [26–65]	0.602
Active tuberculosis history, n (%)	0	0	N/A
Chest computed tomography, n (%)			
Fibrotic changes	6 (13)	5 (7.7)	0.521
Latent tuberculosis treatment after LT, n (%)	4 (8.7)	0	0.027
Nodules on CT after LT, n (%)	20 (43.5)	32 (49.2)	0.569
Excitus, n (%)	8 (17.4)	6 (9.2)	0.452

SD: standard deviation, n: number, TST: tuberculin skin test, IGRA: interferon-gamma release assay, LT: liver transplantation, CT: computed tomography, N/A: not applicable.

**Table 3:** Information about patients who received latent tuberculosis infection treatment.

	Case I	Case II	Case III	Case IV
Age	54	57	70	71
Gender	Male	Male	Female	Female
Transplantation donor type	Cadaveric	Living	Living	Living
Indication	Alcohol	Hepatitis	Cryptogenic	Cryptogenic
Follow-up duration, months	25	11	44	42
IGRA	Positive	Positive	Positive	Positive
TST	22 mm	13 mm	14 mm	17 mm
TB contact	No	No	No	No
CT findings	Nodules	Fibrotic changes	Nodules	Fibrotic changes
LTBI period, months	2	2	1	2
Cause for treatment cessation	Gastrointestinal symptoms	Liver enzyme elevation	Gastrointestinal symptoms	Liver enzyme elevation
Complications	-	Biliary stricture	Hiatal hernia	-

TST: tuberculin skin test, IGRA: interferon-gamma release assay, CT: computed tomography, TB: tuberculosis.

## Data Availability

No new data were created.

## Abbreviations

CT	computed tomography
IGRA	interferon-gamma release assay
LTBI	latent tuberculosis infection
n	number
SD	standard deviation
SOT	solid organ transplantation
TB	tuberculosis
TST	tuberculin skin test
